# Dual focused coherent beams for three-dimensional optical trapping and continuous rotation of metallic nanostructures

**DOI:** 10.1038/srep29449

**Published:** 2016-07-08

**Authors:** Xiaohao Xu, Chang Cheng, Yao Zhang, Hongxiang Lei, Baojun Li

**Affiliations:** 1 State Key Laboratory of Optoelectronic Materials and Technologies, School of Materials Science and Engineering, Sun Yat-Sen University, Guangzhou 510275, China

## Abstract

Metallic nanoparticles and nanowires are extremely important for nanoscience and nanotechnology. Techniques to optically trap and rotate metallic nanostructures can enable their potential applications. However, because of the destabilizing effects of optical radiation pressure, the optical trapping of large metallic particles in three dimensions is challenging. Additionally, the photothermal issues associated with optical rotation of metallic nanowires have far prevented their practical applications. Here, we utilize dual focused coherent beams to realize three-dimensional (3D) optical trapping of large silver particles. Continuous rotation of silver nanowires with frequencies measured in several hertz is also demonstrated based on interference-induced optical vortices with very low local light intensity. The experiments are interpreted by numerical simulations and calculations.

Metallic nanostructures, including nanoparticles and nanowires, are actively researched because of their unique physical properties, which originate from surface plasmon resonance^1^,^2^. Suitable methods to control the positions and movements of the metallic nanostructures can be beneficial to fully exploiting their capabilities. Among the possible methods, optical tweezers has naturally become a first choice because it provides a non-contact and versatile route to trap metallic nanostructures via optical force or to rotate them via optical torque^3^. Until now, the 3D optical trapping of Rayleigh metallic nanoparticles (with diameter *D* ≪ *λ*, where *λ* is the wavelength of light in a vacuum) has been realized using conventional optical tweezers consisting of a single focused beam^4^,^5^,^6^, and this technique has facilitated experiments in areas including biotechnology^7^,^8^,^9^,^10^, nanolithography^11^, acoustics^12^, and nanophotonics^13^,^14^. For example, optically trapped Rayleigh particles have been used to heat attached DNA to tune its binding kinetics^9^, to control polymerization reactions to fabricate polymer nanostructures^11^, to investigate acoustic vibrations away from the substrate^12^, and to enhance surface-enhanced Raman scattering signals^14^. Compared to Rayleigh metallic particles, larger metallic particles have special advantages, including their larger scattering cross-section, their support of higher-order multipoles, their ability to provide larger areas with which to attach biomolecules or cells, and their toxicity for humans^15^; these features are particularly valuable for current studies on biological imaging^16^, plasmon coupling^16^ and cancer therapy^15^. However, the conventional optical tweezers are not always successful in trapping the large metallic particles because the repulsive force (*i.e.,* radiation pressure) caused by the significant scattering and absorption of metallic particles^3^,^17^ increase faster than the attractive force with the particle size. To our knowledge, metallic particles with a diameter of about 250 nm are the largest particles that have been trapped in three dimensions by conventional optical tweezers so far^5^,^6^,^18^,^19^,^20^. Larger metallic particles with diameters of 0.5–3 μm were optically confined only in the transverse region by shaping the Poynting vector distribution of light^21^,^22^. Additionally, several methods based on conventional optical tweezers configurations have been applied to continuously rotate rod-like metallic nanostructures (*i.e.,* metallic nanowires) via the transfer of photon spin or orbital angular momentum^23^,^24^,^25^,^26^. These advances pave the way for metallic nanowires to serve as active elements in next-generation nanomachines, such as fluid-stirring bars in microfluidic devices. However, such methods are usually performed around the light focus, where the local light intensity is extremely high^23^,^24^,^25^,^26^. In this case, the temperature inside the nanowires will be greatly increased, which is liable to damage the nanowires or result in additional heating effects such as fluid convection or the formation of vapor bubbles^3^,^27^. Therefore, the continuous optical rotation of metallic nanowires with low light intensity remains challenging.

Dual beam trap, comprising two counter-propagating coaxial beams, is considered to be a specific trapping geometry that can effectively counteract the radiation pressure^28^. Particularly, when the two beams are tuned to be coherent, axial trapping stability can be considerably enhanced due to the sharp gradient field generated by interference, as theoretically predicted by previous works^29^,^30^. Inspired by these findings, in this work, we utilize dual focused coherent beams as optical tweezers to trap and manipulate metallic nanostructures in water. 3D optical trapping of large metallic particles is realized using a silver nanoparticle with a diameter as large as 800 nm, which noticeably expands size of metallic particles trapped previously by conventional optical tweezers. More importantly, we find that two noncoaxial coherent beams can induce an optical vortex. Based on the interference-induced optical vortex, continuous rotation of a silver nanowire with a diameter of 330 nm and a length of 2.1 μm is demonstrated with a very low local light intensity.

## Experimental Sections

### Experimental setup

**Figure 1:**
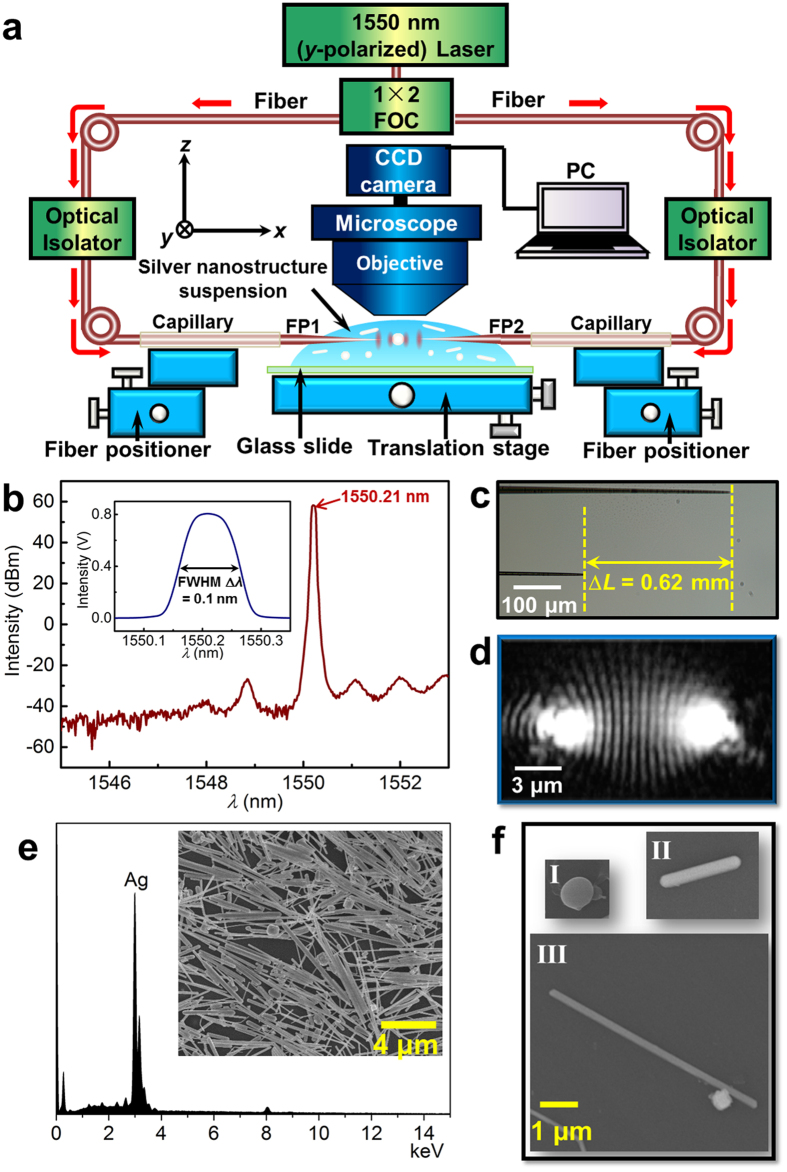
(**a**) Experimental setup of the dual focused coherent beams trapping and manipulation system. FOC, fiber optical coupler; FP1, fiber probe 1; FP2, fiber probe 2. (**b**) Spectral distribution for the 1550 nm laser used in the experiment. The inset shows the full width at half maximum Δ*λ* = 0.1 nm of the light peak. (**c**) Optical microscope image used to measure the length difference Δ*L* of the two fibers. The other ends are aligned. (**d**) Interference pattern generated using the two coherent beams output from FP1 and FP2. (**e**) Energy spectrum and SEM image (inset) of the synthesized silver nanostructures. (**f**) SEM images of the silver nanostructures used in the experiment. I, silver particle (diameter, 800 nm). II, silver nanowire (diameter, 330 nm; length, 2.1 μm). III, silver nanowire (diameter, 230 nm; length, 6.2 μm). The particle near the nanowire in inset III is a silver particle that was attached on the substrate.

Our experimental setup is shown in Fig. 1a. A computer-interfaced optical microscope (Union, Hisomet II) equipped with a charge-coupled device (CCD, Sony iCY-SHOT, DXC-S500) camera was used for real-time observation and image/video capture. The magnification, numerical aperture, and working distance of the objective were ×100, 0.73, and 1.0 mm, respectively. A *y*-polarized laser beam (wavelength *λ* = 1550 nm) is split using a 1 × 2 fiber optical coupler (1:1 splitting ratio) and then launched into fiber probes FP1 and FP2 (see Supplementary Fig. S1 for microscopic images). The two fibers were sheathed by glass capillaries and fixed using tunable six-axis fiber positioners (Kohzu Precision Co., Ltd., 50 nm in resolution) in opposite directions. The tips of the probes were immersed in the silver nanostructure suspension that was placed on a translation stage (50 nm resolution). Two optical isolators were used to avoid feedback from the laser beams. A 1550-nm laser was chosen mainly for two reasons. As we know, 1064 nm is a commonly used wavelength for optical trapping of Rayleigh metallic particles because this wavelength is usually above the plasmon resonance wavelength and can enhance the trapping force^3^. Considering the large metallic particles have longer plasmon resonance wavelength compared to the Rayleigh particles, a longer wavelength (e.g., 1550 nm) will be more favorable for trapping these particles. More details about the choice of wavelength for trapping large metallic particles will be discussed in the simulations and calculations section. Also, sufficient interference fringe spacing to axially confine a large metallic particle can be obtained using the wavelength of 1550 nm. Here, it should be pointed out that, to avoid substantial infrared absorption of water, the optical power applied in the experiment is limited to less than 40 mW.

### Fabrication of the fiber probes

The fiber probes were fabricated using a flame-heating technique by drawing commercial single-mode optical fibers (connector type: FC/PC; core diameter: 9 μm; cladding diameter: 125 μm; Corning Inc.). First, the polymer jacket of a fiber was stripped using a fiber stripper to open a window approximately 50 mm long. The bare part of the fiber was then heated for approximately 1 min to reach its melting point. Then, with a drawing speed of 0.2 mm/s applied to the heated region, the diameter of the bare fiber was gradually decreased from 125 to 9 μm with a length of approximately 2.5 mm. Finally, a high drawing speed of 3 mm/s was applied until the fiber was broken, presenting two abruptly tapered tips with similar shapes. To ensure high spatial coherence of the two counter-propagating beams that are output from the tips of FP1 and FP2, the lengths of the two fibers are as similar as possible. However, due to fabrication errors, the lengths of the two fibers slightly differ. To form dual focused coherent beams, the length difference Δ*L* of the two fibers is controlled to less than the coherence length *λ*^2^/Δ*λ* (~2 cm), where Δ*λ* = 0.1 nm is the full width at half maximum (FWHM) of the light peak produced at 1550 nm (Fig. 1b). In our experiment, Δ*L* = 0.62 mm (Fig. 1c); this value is ≪*λ*^2^/Δ*λ*. Thus, the two output beams exhibit good coherence. Figure 1d shows the interference pattern generated by the two coherent beams, as captured using an infrared camera.

### Preparation of the silver nanostructure suspension

The silver nanowires were synthesized using a polyol process^31^; spherical silver particles were also obtained as by products (see Fig. 1e for the energy spectrum and SEM images, and Supplementary Fig. S2 for the absorption spectrum). The synthesized silver nanostructures were diluted in deionized water with ultrasonic treatment for 5 min. The weight ratio of the silver nanostructures to water was approximately 1:1,500. After preparation, the suspension was dripped onto a clean glass slide using a pipette. Under the influence of gravity, the metallic nanostructures in the sample sink to the bottom (*i.e.,* to the surface of the glass slide). Because the surface roughnesses of the ends of the nanowires differ and the ends of both are larger than those of the other parts of the nanowires, one of the nanowire ends will more easily attach to the surface of the glass slide through stronger van der Waals forces, thus providing a pivot for the optical rotation. A more deterministic control over their attachment to the surface of the glass slide can be achieved by carefully controlling ionic strength and pH^32^. Figure 1f shows scanning electron microscope (SEM) images of a silver particle (inset I) and two types of silver nanowires (insets II and III) used in the experiments.

## Results and Discussion

### 3D trapping of a silver particle

**Figure 2:**
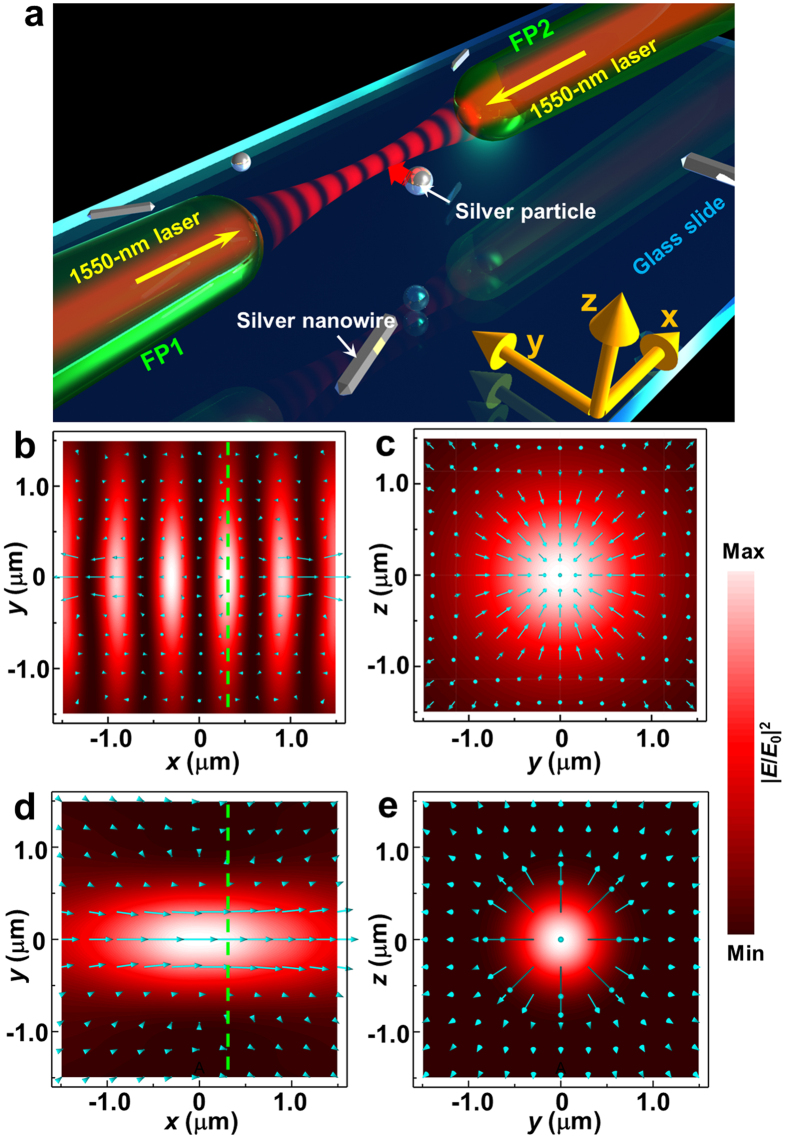
(**a**) Schematic for 3D optical trapping silver nanostructures. Comparison of the simulated electric field intensity distributions and the Poynting vectors (blue arrows) that are generated by dual focused coherent beams (**b**,**c**) and a single Gaussian beam (**d**,**e**). (**b**,**d**) Axial section views. (**c,e**) Transverse section views along the green dashed lines that are shown at *x* = 291 nm in (**b**,**d**), respectively. To clearly show the directions of the Poynting vectors, the scale in (**c,e**) is 10 times larger than that in (**b,d**).

3D trapping of silver particle was conducted by placing the two fiber probes parallel and coaxially, as shown in Fig. 2a. For convenience, we define the *x*-axis as the axial direction of the fiber probe and define the *y*-*z* plane as the transverse plane. To understand the mechanism of 3D trapping, 3D finite-difference time-domain (FDTD) simulations were performed using the commercial software package “FDTD Solutions” (Lumerical, Inc.). In these simulations, FP1 and FP2 were approximated as cones with hemispherical tips (see Supplementary Fig. S3) and were symmetrically situated on the *x*-axis about the original position. The refractive indices of the water and fiber were set to 1.33 and 1.45, respectively. Because the accuracy of the FDTD simulation depends on the size of the mesh grid used, a non-uniform mesh with a maximum grid size of 5 nm was used, which represented a compromise between the accuracy obtained and the computation time required. Figure 2b shows an axial section view of the simulated distribution of the normalized electric field intensity |***E***/***E***_0_|^2^, where ***E*** is the total electric field after focusing and interfering and ***E***_0_ is the incident electric field, for two coherent focused beams that were output from FP1 and FP2 with a tip-to-tip distance *d* = 5 μm (a representative distance in the experiment). The blue arrows show the Poynting vector, which describes the energy propagation and qualitatively indicates the direction of radiation pressure^21^,^33^. When the two beams are propagated in opposite directions, the axial component of the Poynting vector is counteracted in the middle, which will prevent the metallic nanostructures from being pushed away by strong axial radiation pressure. Moreover, the interference fringes, which exhibit a full width at half maximum (~0.25*λ*/*n*_water_, *n*_water_ = 1.33) in the axial direction of much less than the wavelength, provide a sharp gradient field that confines metallic nanostructures in the axial direction. Therefore, metallic nanostructures are expected to be axially trapped between FP1 and FP2 under the action of the axial gradient field that is provided by the interference fringes. Figure 2c shows a transverse section view of the bright interference fringe that is located in the *x* = 291 nm plane (along the green dashed line in Fig. 2b). The laser beam is focused, and the Poynting vector (blue arrows) points inward. Thus, the metallic nanostructures in this region can be attracted into the optical axis under the action of both the gradient force and the radiation pressure. Therefore, it is predicted that a larger metallic particle can also be stably confined in the transverse plane without the action of a repulsive force. It should be pointed out that, for our optical trapping geometry, to ensure that the Poynting vector points inward, the tip-to-tip distance *d* between FP1 and FP2 should be less than 7 μm, such that the laser beams in the trapping region are converging.

For comparison, Fig. 2d shows the axial field distribution, and Fig. 2e shows the transverse field distribution in the *x* = 291 nm plane (along the green dashed line in Fig. 2d); these distributions were generated using conventional optical tweezers comprising a focused Gaussian beam propagating in the positive direction of the *x*-axis. Here, the focused beam is achieved using a high numerical aperture lens (N.A. = 1.0); the focal plane of the beam is set to be *x* = 0. Choosing the *x* = 291 nm plane rather than the focal plane for transverse observation is mainly because that 3D trapping in a single focused beam usually occurs slightly behind the focal plane^34^. Compared to the axial field distribution that is generated by our trapping geometry (Fig. 2b), the axial components of the Poynting vector of the single Gaussian beam in Fig. 2d always point in the direction of light propagation. Therefore, the metallic nanostructures that are present within this single focused Gaussian beam can be easily pushed away along the optical axis because of the forward radiation pressure. Compared to the inward characteristics of the Poynting vector (Fig. 2c), the transverse components of the Poynting vector in Fig. 2e are outward because beams behind the focal plane are diverging; therefore, the transverse trapping force is significantly weakened because of the destabilizing effects of the outward radiation pressure, and the optical trapping becomes unstable.

**Figure 3:**
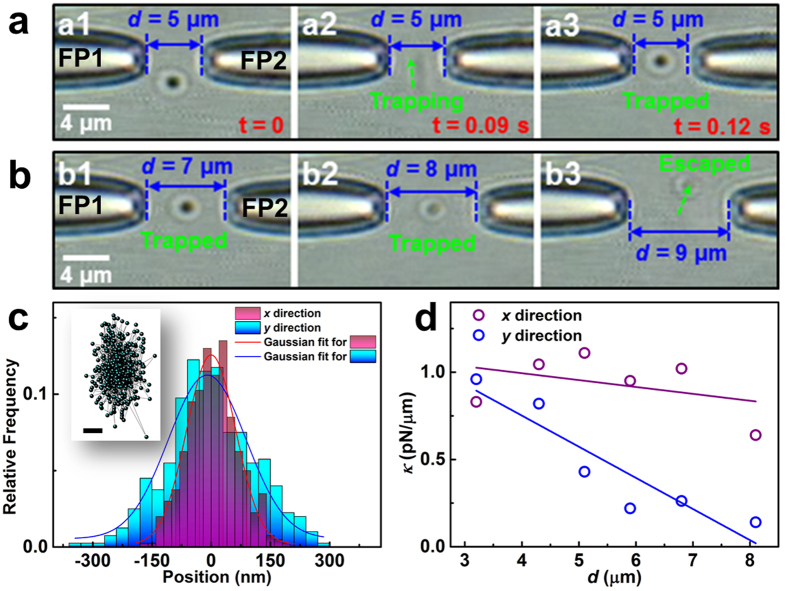
Optical trapping of a silver particle (diameter, 800 nm) using dual focused coherent beams. (**a**) Optical trapping images obtained using a tip-to-tip distance *d* = 5 μm between FP1 and FP2. (**b**) Optical trapping images obtained using various values of *d*. (**c**) Relative frequency of the trapped particle. Lines are Gaussian fits of the data. The inset depicts the corresponding position as a function of time (time step, 30 ms); scale bar, 100 nm. (**d**) The trapping stiffness *κ* as a function of the tip-to-tip distance *d* for the trapped particle. Lines are linear fits of the data.

To verify the simulation results and to demonstrate the trapping ability of the light beam, a silver particle of diameter 800 nm (as shown in inset I of Fig. 1f) is used, and the axial tip-to-tip distance between FP1 and FP2 is set as *d* = 5 μm. The total optical power of the 1550-nm laser is fixed at 10 mW. Figure 3a shows sequential experimental snapshots. After activation of the laser, the nearby particle (*t* = 0, Fig. 3a1) moved toward the fiber tips (*t* = 0.09 s, Fig. 3a2) and was finally stably trapped by the two tips (*t* = 0.12 s, Fig. 3a3). The interference pattern is not visible because a short wave-pass filter was inserted between the CCD camera and the microscope objective. To observe the stability of the trapped particle, the distance *d* was then gradually increased by moving FP2 in the +*x* direction. When the distance *d* was increased to 7 μm (Fig. 3b1) or 8 μm (Fig. 3b2), the particle remained stably trapped. However, at *d* = 9 μm, the trapped particle escaped (Fig. 3b3). The particle escaped because the transverse Poynting vectors tend to point outward.

To determine the probability of trapping, the position fluctuations of the trapped 800-nm silver particle were recorded using automatic tracking software and analyzed. Figure 3c shows the relative frequency of the centroid of the trapped particle at different positions for *d* = 5 μm. The position distributions present Gaussian-like trends, and the full widths at half maximum of the Gaussian distribution are 81 nm in the *x* direction and 142 nm in the *y* direction. The anisotropic fluctuation occurs because the trapping forces in the *x* and *y* directions are derived from the interaction of the particle with the different optical fields, as shown in Fig. 2b,c. To quantify the trapping stability, the trapping stiffness, *κ*, which is related to the position fluctuation of the silver particle, was calculated using the equipartition theorem^35^: *κ* = k_B_*T*/<*ξ*^2^>, where k_B_ is Boltzmann’s constant, *T* is the temperature reported in Kelvin, and <*ξ*^2^> is the variance of the particle position. Figure 3d shows the trapping stiffness *κ* as a function of the tip-to-tip distance *d*. *κ* decreases with increasing *d* and lies in the range of 0.1 to 1 pN/μm. Moreover, the value of *κ* in the *x* direction is larger than that in the *y* direction, indicating that the interference-induced optical gradient force is stronger in the *x* direction. Supplementary Fig. S4 shows the stable movement of the trapped particle in both the transverse and axial directions. The trapping stiffness can also be increased by increasing the optical power to enhance the optical force that acts on the silver nanostructures. However, the power used in the experiment is limited by the absorption of water. Further experimental results show that when the optical power is increased to greater than 50 mW, bubbles are produced^36^. Consequently, the stability of the trapping will be disrupted.

### Continuous rotation of a silver nanowire

**Figure 4:**
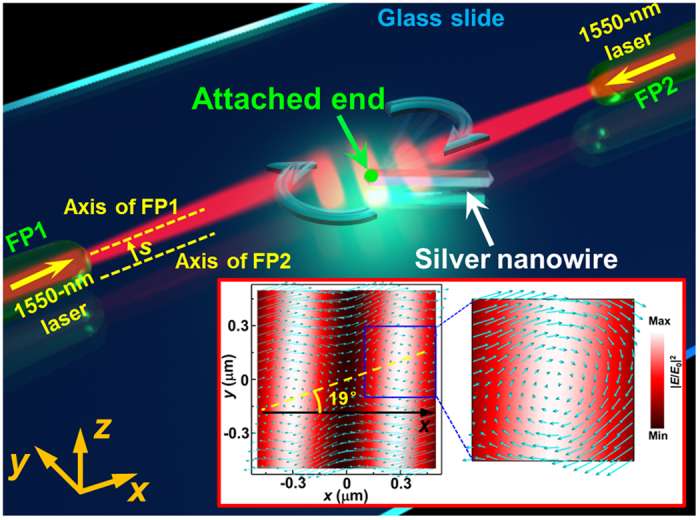
Schematic of the continuous rotation of a silver nanowire. The inset shows the simulated |***E***/***E***_0_|^2^ distribution and the optical vortex that are generated by two noncoaxial coherent beams with an axial distance *d* = 32.8 μm and a transverse distance *s* = 1.0 μm.

Using the same experimental setup, continuous rotation of silver nanowires was also performed. Figure 4 schematically depicts the rotation of a silver nanowire with respect to the end that is attached to a glass slide. In contrast to the geometry used for trapping large silver particles, FP1 and FP2 are not placed coaxially here; instead, they are placed at a transverse distance *s* in the *y* direction between their axes. In this case, optical vortices are formed by the interference between the two non-coaxial coherent beams. As an example, the inset shows the simulated |*E*/*E*_0_|^2^ distribution and the Poynting vector obtained at *d* = 32.8 μm and *s* = 1.0 μm. The Poynting vector is vortex-like in the bright regions, and the centers of the optical vortices are aligned in the 19° direction (yellow dashed line) with respect to the +*x* axis. Therefore, when the attached end of a nanowire is in a vortex, the interaction between the nanowire and the optical vortex will generate an optical torque that rotates the nanowire. It should be emphasized that, these optical vortices are the result of the coherent superposition of the two non-coaxial counter-propagating beams. For comparison, Supplementary Fig. S5 depicts the case for two incoherent beams, for which no optical vortex is formed.

**Figure 5:**
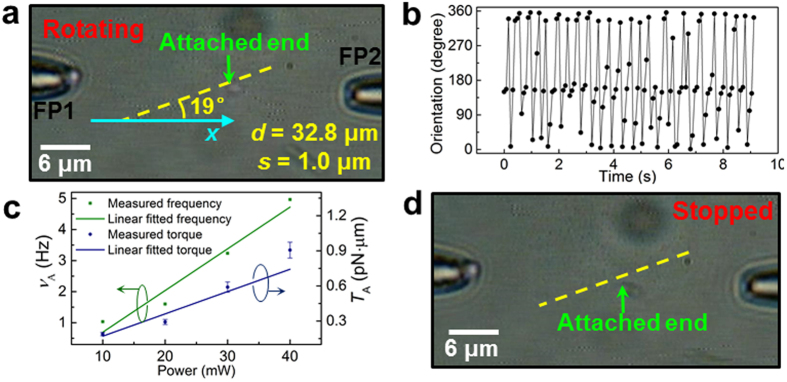
Continuous rotation of a silver nanowire (diameter, 330 nm; length, 2.1 μm). (**a**) Optical microscope image of a rotating nanowire. (**b**) Time trajectory of the nanowire orientations. (**c**) Average rotation frequency *ν*_A_ and the estimated averaged optical torque *T*_A_ as functions of optical power. The lines presented are linear fits. (**d**) An example illustrating that when the nanowire is removed from the optical vortex region, the optical rotation stops.

The rotation experiment was started by launching the laser beam into the two non-coaxial fiber probes (*d* = 32.8 μm, *s* = 1.0 μm). Optical vortices were consequently formed along the yellow dashed line that is shown in the inset of Fig. 4. Then, a silver nanowire of diameter 330 nm and length 2.1 μm (shown in inset II of Fig. 1f) with one end attached to the glass slide via van der Waals forces (see the Methods section for details) was selected and positioned near the optical vortex region by moving the translation stage. After the attached end arrived near an optical vortex, the nanowire began to rotate about the attached end under the action of the optical torque. Figure 5a shows an optical microscope image of the nanowire rotating at an average rotation frequency *ν*_A_ of 3.2 Hz with a total optical power of 30 mW (the image was extracted from Supplementary Video S1). The green arrow indicates the attached end of the nanowire, and the yellow dashed line indicates the alignment direction of the centers of the optical vortices. The silver nanowire was rotated clockwise in a continuous manner, as shown in Fig. 5b. The rotation occurred clockwise due to the interaction of the silver nanowire with the clockwise optical vortex (see the inset of Fig. 4). The average rotation frequency *ν*_A_ can be controlled by changing the optical power as shown in Fig. 5c (green dots and line). *ν*_A_ increases linearly with increasing optical power (from approximately 1 to 5 Hz as the power increases from 10 to 40 mW).

During the rotation, the motion of the silver nanowire is dominated by the competition between the optical torque *T* and the viscous torque *T*_vis_. Because *T* and *T*_vis_ equilibrate almost instantaneously for low Reynolds numbers^37^, the optical torque can be described by^38^


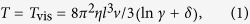


where *η* = (8.0 ± 0.6) × 10^−4^ Pa·s (after taking into account the water heating affects, see Discussion section for temperature increment estimation) is the dynamical viscosity of water, *ν* is the instantaneous rotation frequency of the nanowire, *δ* is the end-correction (as given in ref. 39) as a polynomial expression of (ln 2*γ*)^−1^, and *l* = 2.1 μm and *γ* = 6.7 are the length and aspect ratio of the nanowire, respectively. By replacing the instantaneous rotation frequency *ν* with the average rotation frequency *ν*_A_, the averaged optical torque *T*_A_ was obtained (Fig. 5c, blue dots and line). The *T*_A_ is linearly scaled with the optical power and is approximately 0.1 pN·μm. When the silver nanowire is unable to sufficiently interact with the optical vortex, it cannot be rotated. For instance, the attached end shown in Fig. 5d was moved away from the yellow dashed line by moving the translation stage, and the rotation stopped accordingly. Note that it is necessary to fix the end of the nanowire for continuous rotation; otherwise, an unfixed nanowire will orbit around the vortex (see Supplementary Video S2).

### Simulations and calculations of the trapping

**Figure 6:**
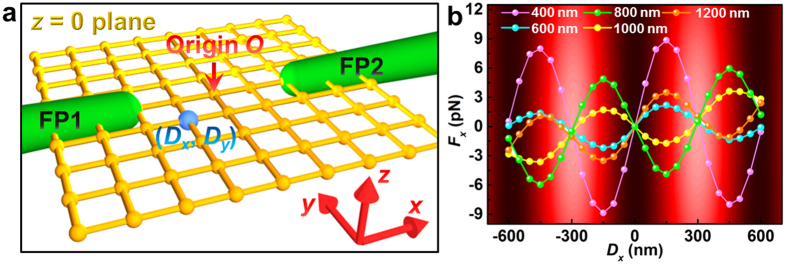
(**a**) The model used for the 3D FDTD simulation. (**b**) The calculated axial optical force *F*_x_ as a function of *D*_x_ for different particle diameters. The interference pattern background (see Fig. 2b) is used to indicate the position of *D*_x_ with respect to the fringes.

To numerically illustrate the trapping performance, the optical force exerted on the silver particle was calculated using the Maxwell stress tensor method. This universal calculation method is suitable for structures with arbitrary shapes and refractive indices. The optical force exerted on the silver particle is given by^27^


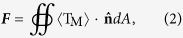


where the integration is performed over the closed surface *A* surrounding the structure, 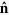 is the unit vector outward normal to the surface *A*, and 〈T_M_〉 is the time-averaged Maxwell stress tensor which can be expressed by


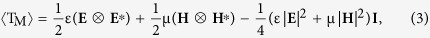


where ⊗ denotes the dyadic product, **I** is the unit dyadic, the superscript * indicates complex conjugation, and ε and μ are the relative permittivity and permeability of the surroundings, respectively. The electric and magnetic field components (**E** and **H**, respectively), which include the multiple scattering effects between the incident field and the metallic nanostructures, can be obtained directly from the FDTD simulation data. Figure 6a shows the simulation model. In addition to the models of fiber probes described above, the silver particle was approximated as a sphere and was situated in the *z* = 0 plane; displacements of its midpoint in the *x* and *y* directions are denoted *D*_*x*_ and *D*_*y*_, respectively. The total optical power is set to 10 mW, which is consistent with the value used in our experiment. The refractive index of the silver particle is adopted from experimental data reported by Johnson and Christy^40^.

Figure 6b shows the axial optical force *F*_x_ exerted on particles with different diameters (400, 600, 800, 1000, and 1200 nm) as a function of *D*_*x*_ for *D*_*y*_ = 0 and *d* = 5 μm. In this figure, the interference fringe background derived from Fig. 2b clearly indicates the position *D*_x_ with respect to the interference pattern. For each particle, the force *F*_x_ changes periodically with *D*_x_ because the axial radiation pressure is almost counteracted along the optical axis and an interference-induced gradient force acts mainly on the particle. Trapping occurs where *F*_*x*_ is zero with a negative derivative; thus, the particles with diameters of 400, 1000, and 1200 nm will be drawn towards the bright fringe region while the particles with diameters of 800 and 1000 nm will be predicted to be trapped in the dark fringe regions. This size-dependent optical trapping can be used for sorting of particles according their size^41^. When a silver particle is trapped in the dark fringe regions, the local field intensity acting on the particle will be minimized; thus, this axial trapping position is beneficial for suppressing the heating effect of the trapped particle.

**Figure 7:**
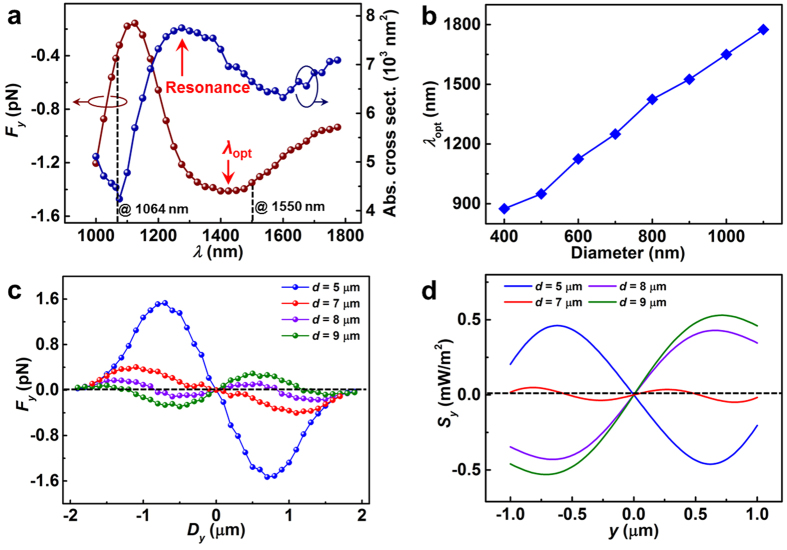
(**a**) The absorption cross-section and the transverse force *F*_*y*_ as a function of wavelength for a silver particle of diameter 800 nm. (**b**) The optimal wavelength *λ*_opt_ as a function of silver particle size. (**c**) The transverse force *F*_*y*_ exerted on silver particles of diameter 800 nm obtained using various values of *d*. (**d**) The simulated transverse Poynting vector *S*_*y*_ obtained using various values of *d*.

It should be pointed out that, when an Ag particle is trapped at intensity minima, it does not mean that the gradient force acting on this particle is repulsive but predicts the resultant force exerted on the particle is zero with a negative derivative at intensity minima. For example, the 800 nm silver particle is predicted to be trapped at the dark fringe region (as indicated in Fig. 6b). This is mainly because that the particle can overlap two bright fringes and thus under the action of two gradient fields, the resultant force exerted on the particle is zero with a negative derivative at intensity minima. However, the gradient force acting on the particle is attractive, which is explained by the following analyses. The direction of the gradient force is determined by the trapping wavelength with respect to the spectral position of the resonance wavelength^20^,^33^,^42^. Particularly, the gradient force is attractive for the trapping wavelengths on the red side of the resonance wavelength and is repulsive for the trapping wavelengths on the blue side of the resonance wavelength. For the 800 nm silver particle, its absorption spectrum in Fig. 7a (blue line) shows that the trapping wavelength (*λ* = 1550 nm) is on the red side of the resonance wavelength (*λ*_res_ = 1275 nm) and thus the gradient force acting on the particle is attractive.

For the transverse direction, the optical wavelength used can greatly affect the trapping force exerted on metallic particles^43^,^44^. In Fig. 7a, the dark red line shows the calculated value of *F*_y_ as a function of wavelength for a silver particle of diameter 800 nm that is located at *D*_x_ = 0 and *D*_y_ = 500 nm. The optimal wavelength *λ*_opt_ for trapping the silver particle is 1425 nm, and the largest trapping force *F*_y_ is −1.43 pN. Nevertheless, the value of *F*_y_ obtained at 1550 nm remains strong (*F*_y_ = −1.27 pN); this force is approximately 3 times as large as that (*F*_y_ = −0.42 pN) obtained at 1064 nm (a wavelength that is commonly used in optical manipulation^33^,^45^). Figure 7b shows the optimal wavelength *λ*_opt_ for trapping other large particles. Longer wavelengths are recommended for trapping larger silver particles.

To numerically study the relationship between transverse trapping ability and the tip-to-tip distance *d*, the transverse forces *F*_*y*_ acting on the silver particle of diameter 800 nm were calculated for various values of *D*_*y*_ and *d* (Fig. 7c). The force *F*_*y*_ is sensitive to *d*. When *d* = 5 μm (blue line) and 7 μm (orange line), the forces *F*_*y*_ are always attractive; thus, the particle can be stably trapped. As *d* is increased to 8 μm (violet line), the force *F*_*y*_ fluctuates and remains attractive at some locations (e.g., |*D*_*y*_| > 800 nm). When *d* = 9 μm (green line), the force *F*_*y*_ nearly becomes repulsive; thus, the optical trapping will be disrupted. These results are consistent with our experimental results (see Fig. 3b). The trapping performance obtained with increasing *d* is strong relative to the distribution of the Poynting vector. Figure 7d shows the distribution of the simulated transverse components of the Poynting vector *S*_y_ for various values of *d*. *S*_y_ is inward (corresponding to converging beams) for small values of *d* (e.g., *d* = 5 μm), but tends to become outward (corresponding to diverging beams) at higher values of *d*. In particular, when *d* = 9 μm, a strong outward *S*_y_ is produced, making stable trapping difficult (see green line in Fig. 6e). From Fig. 7c,d, it is easy to find that more convergent beams produce more robust trapping.

### Simulations and calculations for continuous rotation

**Figure 8:**
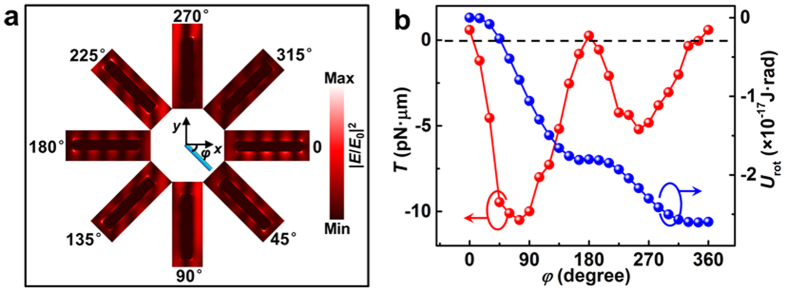
Simulated and calculated results for continuous rotation. (**a**) Simulated |***E***/***E***_0_|^2^ distribution for a silver nanowire (diameter, 330 nm; length, 2.1 μm) at various orientations *φ*. (**b**) The calculated value of the optical torque *T* (red symbols) exerted on the nanowire and the corresponding rotational potential *U*_rot_ (blue symbols) as a function of *φ*.

To explain the continuous rotation phenomena and estimate the optical torque induced by the two noncoaxial coherent beams, a serial of simulations and calculations was performed. Figure 8a shows simulated |*E*/*E*_0_|^2^ distributions around the nanowire (diameter, 330 nm; length, 2.1 μm; consistent with the experiment) with angle *φ*** = **0°, 45°, 90°, 135°, 180°, 225°, 270°, and 315°, where *φ* denotes the angular orientation with respect to the positive *x* axis. The electric field intensity and therefore the Maxwell stress tensor distributions around the nanowire are asymmetric, resulting in an optical torque that rotates the wire. The optical torque *T* exerted on the nanowire is given by^38^,^46^


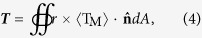


where ***r*** is the position vector with respect to the rotation axis. The +*z* direction denotes the positive direction of the optical torque. Figure 8b shows the calculated optical torque *T* (red line) acting on the nanowire as a function of the angle *φ* with a total optical power of 10 mW. Except for the orientation around *φ* = 0° and 180° (360° is equivalent to 0°), the value of *T* is negative. The minimum torque is approximately −10.5 pN·μm, and the absolute value is much larger than the maxima (~0.6 pN·μm). The small positive torque was mainly caused by interactions with the optical vortices that are located in other bright fringe regions. To demonstrate the continuous rotation, the rotational potential *U*_rot_ (blue line) was calculated by integrating the optical torque with respect to the orientation angle *φ*. The potential *U*_rot_ generally decreases with increasing *φ*, thus causing the nanowire to rotate clockwise to decrease the potential. Two potential wells exist at approximately 165° and 345° and were caused by the resistance effects of the positive torque at approximately 180° and 360° (*i.e.,* 0°). However, the two potential wells are very shallow, with depths of 7 × 10^−20^ and 2 × 10^−20 ^J·rad, respectively; thus, these wells can easily be overcome by the rotational kinetic energy of the nanowire. Therefore, continuous rotation of nanowires can be realized using dual noncoaxial coherent beams. Based on the above numerical results, the average optical torque *T*_NA_ is calculated to be 3.9 pN·μm according to *T*_NA_ = Δ*U*_rot_/2π, where Δ*U*_rot_ is the difference between *U*_rot_ at *φ* = 0 and 360°. The difference from the experimental value (*T*_A_ ~ 0.3 pN·μm) is mainly induced by wall effects (Faxén’s law) that are related to interactions with the glass slide^47^. Because *T*_NA_ > *T*_A_, the wall effects are expected to enhance the viscous force acting on the nanowire; these results are similar to the reported theoretical and experimental results for a microparticle^47^.

## Discussion

The above experimental and calculated results show that dual focused coherent beams can provide the stable 3D optical trapping of large silver particles and the continuous rotation of silver nanowires. The 3D trapping is attributed to the sharp interference fringes (Fig. 2b) and to the inward transverse Poynting vector (Fig. 2c). In addition, the 1550-nm laser used here can provide a stronger transverse trapping force than the 1064-nm laser that is commonly used (Fig. 7a). The key to the continuous rotation of the metallic nanowire is the optical vortex that is induced by the coherent superposition of the two noncoaxial counter-propagating beams and that occurs far from the fiber probes (Fig. 5a) where the local field intensity is extremely low. For the silver nanowire 2.1 μm in length that is rotated continuously at a frequency of a few hertz with an optical power of tens of mW in our experiment, the incident field intensity in the optical vortex is estimated to be approximately 1 × 10^10^ W/m^2^ based on the simulation result shown in the inset of Fig. 4. For comparison, a silver nanowire 2 μm in length rotated at the optical focus based on a photon spin angular momentum transferring mechanism with a comparable rotation frequency and optical power^25^ has an estimated incident field intensity up to approximately 1 × 10^12 ^W/m^2^ (see Supplementary Fig. S6), a value that is much higher than that obtained by our method. The low light intensity required for rotation by our method is attributed to the lateral illumination geometry, which produces a sufficient light-nanowire interaction. When a given metallic nanowire is immersed in water and illuminated by light, the temperature increment Δ*T* inside the nanowire due to its absorption can be evaluated using steady-state model^48^. For the nanowire 330 nm in diameter and 2.1 μm in length and the illumination intensity of 1 × 10^10 ^W/m^2^ in our experiment, Δ*T* ranges from 2 to 9 °C, which only result in a temperature of 27–34 °C inside the nanowire (see Supplementary Fig. S7 and text in the Supplementary Information for calculation details); this temperature is well below the damaging temperature of metallic nanostructure^49^. Note that although water has strong absorption at the 1550 nm wavelength, previous work has proven that water heating-induced flow with an optical power of 40 mW is observable only at the surface of water^50^. But, in our experiment, the optical rotation was operated near the water bottom and thus the heating effects by absorption of water can be negligible.

**Figure 9:**
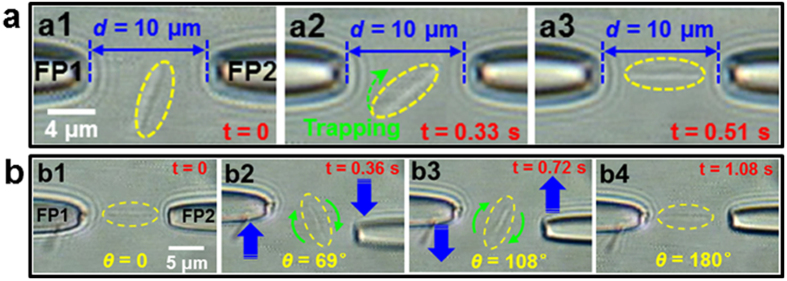
(**a**) 3D optical trapping of a 230-nm-diameter and a 6.2-μm-long silver nanowire (see the inset III of Fig. 1f). The tip-to-tip distance is *d* = 10 μm. (**b**) Consecutive microscopic images for rotating the trapped 230-nm-diameter and 6.2-μm-long silver nanowire from 0 to 180° with a time interval of 0.36 s.

Our previous work demonstrated that a silver wire 600 nm in diameter and 6.5 μm in length can be trapped and rotated using two incoherent optical beams^51^. Axial trapping of the nanowire was achieved using only the counter-direction radiation pressures, which will affect the trapping stability–especially for thin nanowires. Moreover, the trapped nanowire cannot be rotated continuously using this method. These limitations are successfully overcome by the dual focused coherent beams configuration. Because of the high spatial coherence of the dual beams, the axial trapping force acting on the nanowires can be strongly enhanced by the sharp gradient field resulting from the interference fringes, thus stably trap the thin nanowires. As an example, Fig. 9a shows the 3D optical trapping of a silver nanowire 230 nm in diameter and 6.2 μm in length. Once the laser was turned on (Fig. 9a1), the nanowire was moved towards the optic axis and was simultaneously rotated clockwise (Fig. 9a2). Finally, it was stably trapped along the optical axis (Fig. 9a3). According to Fig. 9a3, the balanced orientation of the trapped nanowire is horizontal; in our previous work, the orientation was vertical^51^. This result is mainly explained by the coherent superposition of light, which can produce stronger local electric field intensities than those produced by incoherent superposition (see Supplementary Fig. S8). In this case, the trapped nanowire can be continuously rotated by operating the fiber probes. Figure 9b shows consecutive images of a trapped silver nanowire rotating from 0 to 180° with a regular time interval of 0.36 s. The angular orientation of the nanowire with respect to the negative *x* direction is defined as *θ*. Firstly, the trapped nanowire was oriented at *θ* = 0 (Fig. 9b1). Then, by increasing the transverse distance between the FP1 and the FP2, the nanowire was rotated clockwise due to the asymmetrical field distribution (Fig. 9b2). As *θ* is larger than 90°, by decreasing the transverse distance, the rotation direction of the nanowire kept clockwise (Fig. 9b3). Finally, the nanowire was oriented at *θ* = 180° (Fig. 9b4) and returned to the initial trapping state. Continuous rotation of the trapped nanowire can be realized by periodically repeating this process. In addition, the stable position and orientation trapping of silver nanowires enables the assembly of structures (see Supplementary Figs S9 and S10), that can be used as nanoscale photonic circuit elements such as plasmonic routers and multiplexers^52^,^53^ or plasmonic modulators^53^,^54^. Therefore, our strategy also enables all-optical control of rod-like metallic structures.

## Conclusions

From theoretical and experimental viewpoints, we have demonstrated optical tweezers that are capable of the three-dimensional trapping of large silver particles and of the continuous rotation of silver nanowires using dual focused coherent beams. The ability to trap large metallic particles would facilitate new experiments on the plasmon coupling of higher-order multipolar contribution, a regime that has yet to be studied in detail. Another attractive and promising avenue of research is to utilize the optically trapped large metallic particles for the fundamental researches on cancer therapy. Because of the large scattering and absorption cross-section of these particles, they can be employed as transportable probes to detect cancer cells via surface-enhanced Raman scattering and kill the cells via particle heating. The optical rotation of metallic nanowires is an important technique that enables the operation of nanomachines in applications such as microfluidics. To induce a stable fluid by rotational movement without introducing additional heating effects, which are usually unpredictable, the intensity of the light that is used to rotate the metallic nanowire should be minimized. The optical rotation of silver nanowires demonstrated here was performed with an extremely low incident field intensity (approximately 1 × 10^10^ W/m^2^), significantly decreasing the heating effects produced. Thus, such optical tweezers can provide new opportunities in various scientific fields including nanophotonics, biophotonics, and optofluidics.

## Additional Information

**How to cite this article**: Xu, X. *et al*. Dual focused coherent beams for three-dimensional optical trapping and continuous rotation of metallic nanostructures. *Sci. Rep.* **6**, 29449; doi: 10.1038/srep29449 (2016).

## Supplementary Material

Supplementary Information

Supplementary Video S1

Supplementary Video S2
