# Supplementary material for: Dual focused coherent beams for three-dimensional optical trapping and continuous rotation of metallic nanostructures
Source: Sci Rep. 2016 Jul 8;6:29449. doi: 10.1038/srep29449 (PMC4937446; doi:10.1038/srep29449)
Supplement: Supplementary Information [file srep29449-s1.doc]

**Supplementary information**

# Dual-beam focused coherent beams for three-dimensional optical trapping and continuous rotation of metallic nanostructures

XiaohaoXu, Chang Cheng, Yao Zhang, Hongxiang Lei^*^, and Baojun Li^*^

State Key Laboratory of Optoelectronic Materials and Technologies, School of Materials Science and Engineering, Sun Yat-Sen University, Guangzhou 510275, China

*Corresponding author: leihx@mail.sysu.edu.cn, stslbj@outlook.com

**Supplementary Videos**

Video S1 showing that a silver nanowire of diameter 330 nm and length 2.1 μm was rotated continuously at an average rotation frequency *ν*_A_ of 3.2 Hz with a total optical power of 30 mW.

Video S2 showing that an unfixed silver nanowire orbits around the optical vortex.

**Supplementary Figures**


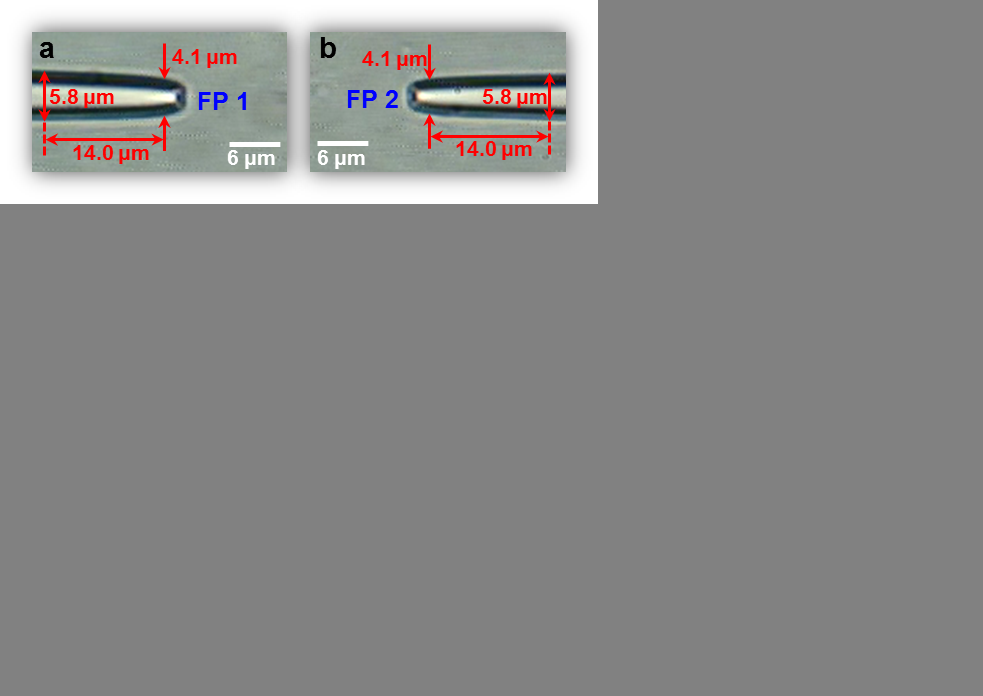


**Figure S1** Optical microscopic images of tapered FP1 and FP2 with quasi-hemispheroidal tips. The FP1 and FP2 are gradually tapered from 5.8 to 4.1 μm within a length of 14 μm.


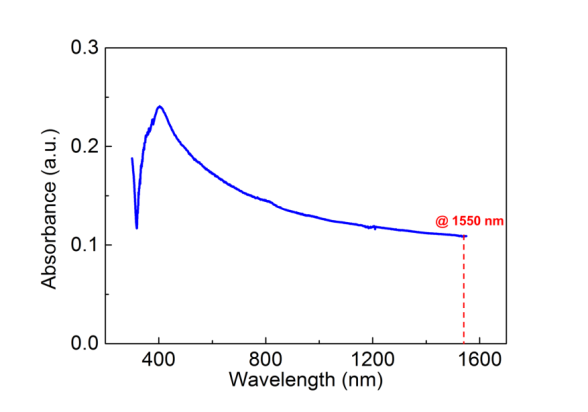


**Figure S2** Absorbance of an ensemble of silver nanostructures in water.


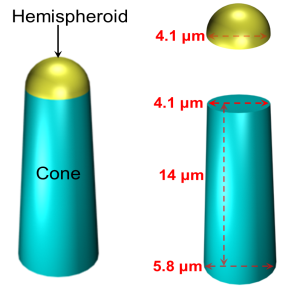


**Figure S3** Simulation model for the fiber probe, which is approximated as a cone with a hemispherical tip.


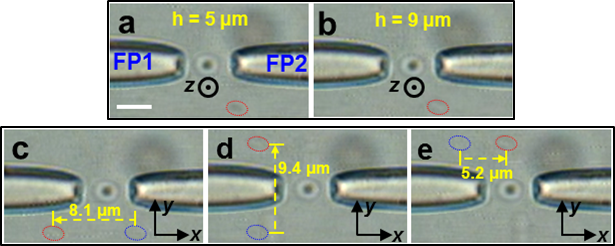


**Figure S4** (a,b) 3D move of the trapped 800 nm silver particle in the *z* direction and (c−e) in the *x*-*y* plane. Red circles indicate the location of a particle that is stationary as a marker. Blue circles in (c−e) indicate the location of the marker in (b−d), respectively. The white scale bar is 4 μm. Initially, the particle was trapped at height *h* = 5 μm from the glass slide with *d* = 5 μm (a). Then, by moving down the translation stage along −*z* direction, the trapped particle is localized at *h* = 9 μm (b). Further moving toward −*x* direction with a distance of 8.1 μm, the trapped particle is moved in the opposite direction (i.e. +*x* direction) (c). Similarly, (d,e) shows the trapped particle is moved 9.4 μm along −*y* direction and then 5.2 μm along −*x* direction, respectively.


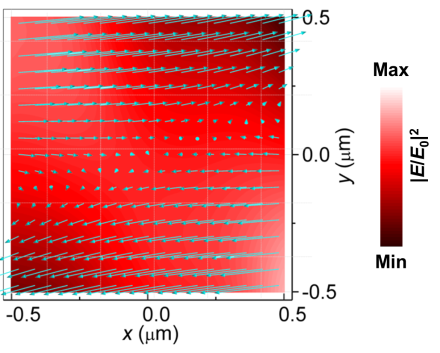


**Figure S5** Distribution of the electric field intensity and the corresponding Poynting vector generated by two noncoaxial incoherent beams. It shows that there is no optical vortex occurred and the direction of the Poynting vector is characterized by simple incoherent superposition of beams.


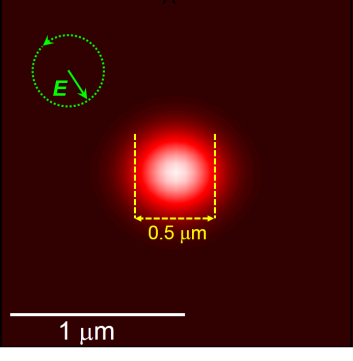


**Figure S6** Simulated spot near the focus for a circularly polarized laser with 800 nm wavelength and a lens with N.A. = 0.7 that are used in ref. 25. The diameter of the formed spot is 0.5 μm. Therefore, for a laser power of tens of milliwatt applied in ref. 25, the light intensity near the focus is calculated to be of the order of 1 × 10^12^ W/m^2^.


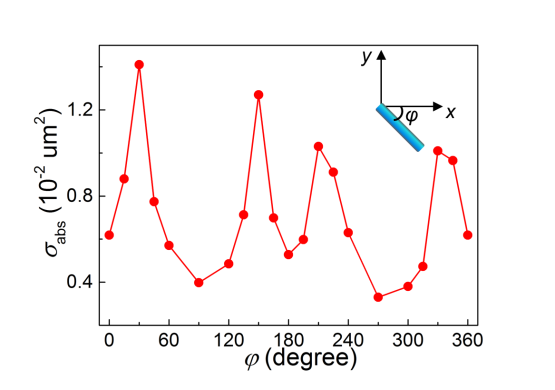


**Figure S7** Calculated absorption cross sections *σ*_abs_ for a silver nanowire (diameter, 330 nm; length, 2.1 μm) at different orientations *ϕ*. The absorption cross sections have an orientation averaged value *σ*_aabs_ = 7.3 × 10^-15^ m^2^, a maximum value *σ*_max-abs_ = 1.4 × 10^-14^ m^2^ and a minimum value *σ*_min-abs_ = 3.3 × 10^-15^ m^2^.


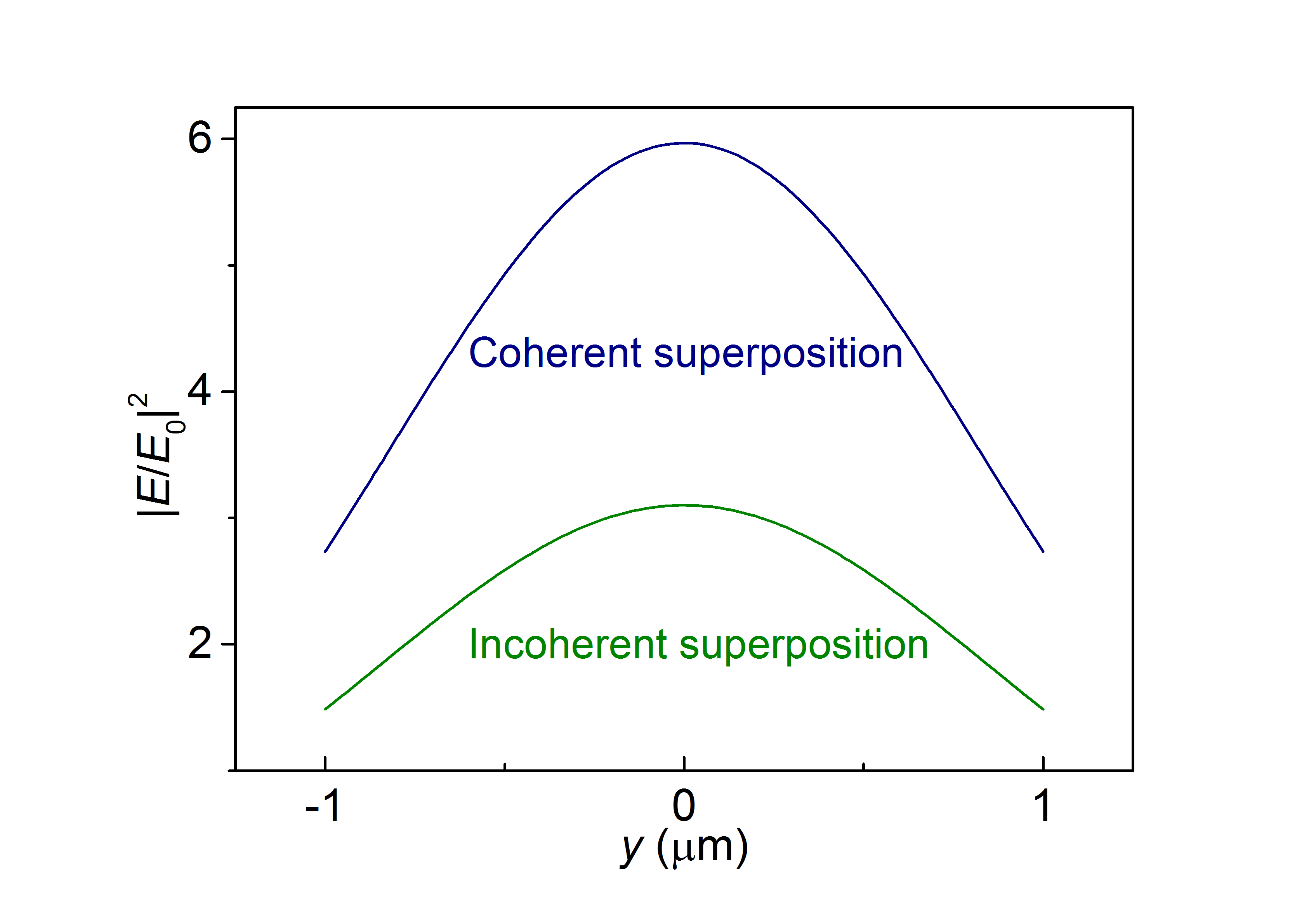


**Figure S8** Transverse electric intensity distribution generated by coherent and incoherent superposition of the two counter-propagated beams.


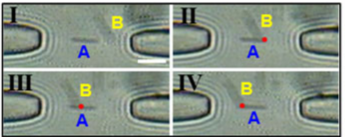


**Figure S9** Optical assembly of branched silver nanowire structures. A 300-nm-diameter and 3.9-μm-long nanowire A was trapped and delivered toward another stuck nanowire B by moving the translation stage along −*x* direction (I). Through assembling A onto B, different shapes of branched structures were constructed (II−IV). The white scale bar is 4 μm.


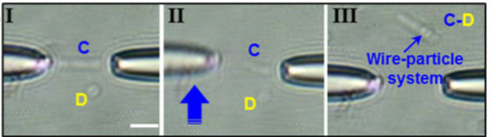


**Figure S1****0** Optical assembly of nanowire-particle system. (I) A 230-nm-diameter and 6.2-μm-long nanowire C was trapped and delivered to a stuck particle D. (II) To show the ability for assembling C to D with a special orientation, the nanowire was firstly reoriented with the angular orientation by moving the left FP. (III) By moving the translation stage, the nanowire C approached to the particle D and finally they were attached together via van der Waals forces. The white scale bar is 4 μm.

**Supplementary Text**

Photothermal Effect of Illuminated Silber Nanowire

For metal nanowires immersed in water and illuminated by light of intensity *I* (*I* is approximately 1 × 10^10^ W/m^2^ in our experiment), the temperature increment under steady-state can be expressed as (see Ref. 47 in the manuscript)

$\Delta T=\frac{I\sigma_{abs}}{4\pi R_{eq}\beta\kappa_{water}}$, (1)

where *σ*_abs_ is the absorption cross section of the wire (see Fig. S7), Req is the radius of a sphere with the same volume as the nanowire, *β* is a dimensionless thermal capacitance coefficient, *κ*_water_ is the thermal conductivity of water (0.6 Wm^−1^K^−1^). *R*_eq_ and *β* can be expressed as

*R*_eq_ = (3*D*^2^*L*/16)^1/3^ (2)

and

*β* = 1+0.96587ln^2^(*L*/*D*), (3)

where *L* is the length of nanowire, *D* is the diameter. For a silver nanowire with *D* = 330 nm and *L* = 2.10 μm, we have *R*_eq_ = 0.35 μm and *β* = 5.72. Thus the average temperature increment is estimated to be ∆*T* = 5 K. Particularly, the maximum and minimum temperature increment are estimated to be ∆*T*_max_ = 9 K and ∆*T*_min_ = 2 K, respectively.
